# Indirect effects of hyaluronic acid applications on the glabella and nasolabial regions: An anatomic evaluation

**DOI:** 10.1097/MD.0000000000042390

**Published:** 2025-05-09

**Authors:** Ayse Gul Kabakci, Dilek Eren, Dursun Murat Bozkir, Çağlar Cengizler, Eda Esra Esen, Yaşam Türközer, Memduha Gülhal Bozkir

**Affiliations:** a Department of Anatomy, Cukurova University, Adana, Turkey; b Dr Dilek Eren Medical Aesthetic Clinic, Adana, Turkey; c Sevgi Eye Center, Seyhan, Turkey; d Biomedical Device Technology Program, Vocational School of Health Services, Izmir Democracy University, Izmir, Turkey.

**Keywords:** anatomy, glabella, hyaluronic acid, nasolabial fold

## Abstract

This study is among the first to systematically evaluate the indirect anatomical effects of hyaluronic acid injections on the glabellar and nasolabial regions. It focuses on changes in wrinkle severity and anatomical parameters to enhance safety and efficacy. In this study, we analyzed pre- and post-treatment photographs of 31 women with a mean age of 45.97 ± 7.10, who received hyaluronic acid treatment. The study was designed retrospectively. Photographs taken before the procedure and one month after treatment were obtained from the clinical archive. Digital anatomical measurements were performed on these images. The anatomical measurements focused on the glabella (including facial convexity, total facial convexity, and facial concavity) and the nasolabial regions (including nasolabial and nasolabial fold angles on both the right and left sides). Wrinkle Severity Rating Scale (WSRS) evaluations were also conducted for both the glabella and nasolabial folds. Evaluations were performed by analyzing photographs taken before, 4 weeks after, and 12 weeks after hyaluronic acid filler application. In the glabellar region, hyaluronic acid injections into the temporal and zygomatic areas significantly reduced wrinkles, especially 12 weeks post-treatment (*P* = .004), indicating an indirect improvement in glabellar wrinkles. A positive correlation between age and wrinkle improvement was found (*r* = 0.601, *P* = .000), suggesting more pronounced effects in older individuals. In the nasolabial region, significant reductions were observed in both the right (*P* < .001) and left (*P* < .001) nasolabial folds post-treatment, with age also correlating with improvement (right: *r* = 0.693, *P* = .000; left: *r* = 0.496, *P* = .005). The S300 and S500 formulations reduced wrinkles, with the S500 showing greater efficacy than the S300. The findings of our study suggest that hyaluronic acid fillers can indirectly reduce the depth of nasolabial folds, and this effect can be achieved particularly when applied using techniques that are suitable for anatomical and vascular structures. This approach may be considered a viable alternative in aesthetic procedures, as it has the potential to enhance treatment safety by reducing the risk of complications associated with direct interventions.

## 1. Introduction

Interventional procedures, whether for diagnostic, therapeutic, or cosmetic purposes, are widely utilized in medical practice. A deep understanding of the anatomy of the targeted area is crucial for the safe and effective execution of these procedures. The facial region, in particular, presents unique challenges due to its dense vascular structures and frequent anastomoses, which may increase the risk of complications during interventions. While the high vascularization can contribute to tissue healing, it also heightens the potential for bleeding. Therefore, for procedures such as hyaluronic acid injections commonly used in facial aesthetic treatments, a comprehensive knowledge of facial anatomy is essential to minimize risks and achieve optimal results.^[1]^

The glabellar and nasolabial regions are critical in facial aesthetics and clinical procedures, but their dense vascular networks and proximity to vital neurovascular structures present significant risks during interventions. The variability of arteries in the glabellar area and the dense vascular supply of the nasolabial region can lead to complications such as bleeding, hematoma, and nerve injury.^[2–5]^ Notably, the supraorbital and supratrochlear arteries in this area exhibit significant variability in their course and branching patterns, which can lead to complications such as bleeding or hematoma formation.^[2]^ Additionally, the superficial branches of the trigeminal nerve in these areas increase the risk of sensory changes or facial asymmetry.^[3–8]^ Additionally, complications in the nasolabial region can arise from the characteristics of the dermal and subcutaneous tissues.^[9]^ These complications highlight the importance of a thorough understanding of the anatomy for safe and effective interventions. Given these risks, our study hypothesizes that applying hyaluronic acid fillers to the temporal and zygomatic regions can reduce complications in the glabellar and nasolabial areas. We hypothesize that applying hyaluronic acid fillers to the temporal and zygomatic areas can provide a beneficial secondary effect on the glabellar and nasolabial regions. So, the primary objective of our research is to anatomically analyze the indirect effects of hyaluronic acid filler applications to the temporal and zygomatic regions on the glabellar and nasolabial areas. We aim to mitigate potential complications from direct hyaluronic acid filler treatments in the glabellar and nasolabial regions by focusing on these indirect methods. This approach allows for a more strategic application of aesthetic treatments, reducing the risk of adverse outcomes associated with direct interventions in these delicate zones. Moreover, our study addresses these concerns by exploring alternative approaches to rejuvenating these high-risk areas. Although direct filler applications are typical, there are no studies investigating the potential benefits of indirect methods in reducing complications. This gap in the literature led to the hypothesis that hyaluronic acid application to the temporal and zygomatic areas may offer a viable solution to reduce complications and improve overall outcomes.

## 2. Methods

### 2.1. Study design

The planned study was a single-center, noninferiority, retrospective study evaluating the effectiveness and safety of hyaluronic acid on the healing of the glabella and nasolabial areas in 31 female subjects. This study was designed as a retrospective study due to specific ethical constraints and logistical challenges that made conducting a prospective study unfeasible. Initiating a prospective study was not possible due to time and resource limitations. Thus, the decision was made to utilize existing clinical data retrospectively. The photographs of the 31 female participants included in our study were selected based on consent forms signed by the participants before the procedure. These forms contained important information regarding the participant’s health status and suitability for the treatment. Collected data consists of the images taken from the clinic archive of individuals who met the inclusion criteria. The study instructions, regulations, and measurements were conducted in accordance with the Declaration of Helsinki. Ethical approval was obtained from the Çukurova University Faculty of Medicine Non-Interventional Clinical Research Ethics Committee on June 2, 2023 (approval number: 134/44). Additionally, the study was supported by the Çukurova University Scientific Research Projects Unit under project number TSA-2024-16787. This study was derived from the TSA-2024-16787 project.

### 2.2. Photos selection criterias

All photos were taken under standardized conditions to ensure consistency and control for variability. Fixed lighting conditions were used, and camera settings (such as ISO, focal length, and aperture) were standardized. Additionally, each patient’s positioning was adjusted to ensure clear visibility of facial features, minimizing the risk of variability between images. Photos used in the study were selected from the clinical archive and included individuals meeting the following criteria:

Informed Consent: Written informed consent for the use of their photos.

Age and Gender: Female patients aged 35 to 55 years.

Hyaluronic Acid Application: Hyaluronic acid application must have been performed (European Polytech Quality [e.p.t.q.®] Lidocaine s300 and s500).

Amount and Location of Application: A total of 4 mL of hyaluronic acid was applied: 1 mL temporal and 1 mL zygomatic on the right side, and 1 mL temporal and 1 mL zygomatic on the left side. The application was made to the zygoma and temporal areas with the same physician, method, and brand (e.p.t.q.® Lidocaine s300 and s500).

Photo Timing: Photos were taken before, 4 weeks after, and 12 weeks after the hyaluronic acid application.

Treatment History: No facial treatments (injections or surgery) were applied within 6 months before the first photograph.

### 2.3. Application details of participants

Photos of 31 women treated with the same dose and method were selected for the study. Applications were performed using separate cannulas on both sides of the face. The same brand and concentration of hyaluronic acid filler (e.p.t.q.® Lidocaine S500; JETEMA Co., Ltd.) were used. Additionally, this product was approved by the Food and Drug Administration. Also, each author has stated that there was no commercial support related directly or indirectly to the subject of the study. Our study sample included those who were administered e.p.t.q.® Lidocaine s300 and s500. Patients were separated and analyzed according to age groups. It was determined that those under 40 were applied s300 and those who were applied s500 were 41 years old and over.

### 2.4. Scale and anatomic analysis

Photos from the clinical archive were selected using the observ®520 system. The photos were evaluated using the Wrinkle Severity Rating Scale (WSRS). The WSRS score was assessed before the application, 4 weeks after, and 12 weeks after the application, and was rated as follows:

None (1 point)

Mild (2 points)

Moderate (3 points)

Severe (4 points)

Extreme (5 points)

The WSRS score was interpreted as directly proportional to the degree of wrinkles.

### 2.5. Anatomic parameters

The measurement process was performed using the ImageJ software (version 1.52a), which offers a sensitivity of 1/100 mm. Initially, the distal point of the angle to be measured was identified, and a reference line was drawn from this point to the midpoint of the angle. The angle was then determined by drawing a second line from the midpoint to a third reference point, with the mouse guiding the positioning of the lines. The resulting angle measurements were recorded and compared between the pretreatment and post-treatment images. To ensure consistency and reliability, the same operator conducted all measurements 3 times, minimizing variability.

**Figure 1.**
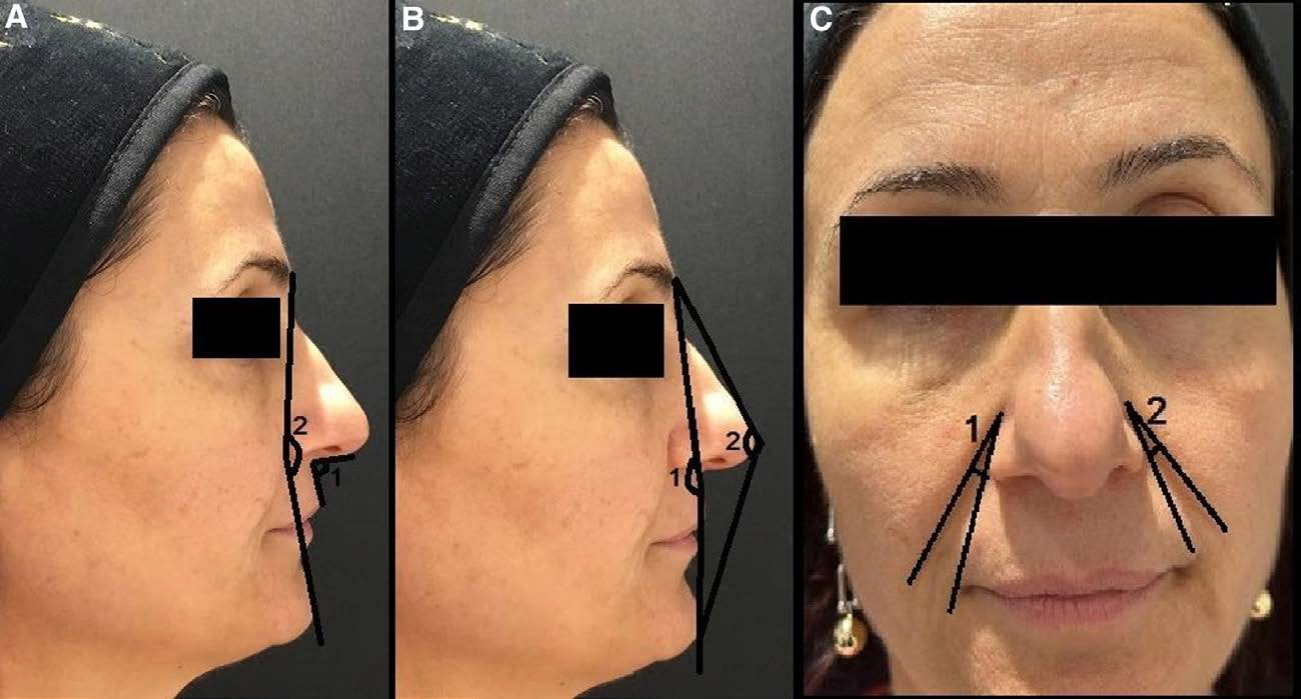
Anatomical landmarks of measurement parameters (A1: Nasolabial angle between the columella, subnasale, and labiale superius; A2: Facial concavity between the glabella, alar rim, and progonion; B1: Facial convexity between the glabella, subnasale, and progonion points; B2: Total facial convexity between the glabella, pronasal, and progonion points; C1: Nasolabial fold angle right side between the highest point of the nasolabial fold and the alar rim; C2: Nasolabial fold angle left side between the highest point of the nasolabial fold and the alar rim).

Facial Convexity: The angle was measured between the glabella, subnasale, and progonion points (Figure 1B1).

Total Facial Convexity: The angle was measured between the glabella, pronasal, and progonion points (Figure 1B2).

Facial Concavity: The angle was measured between the glabella, alar rim, and progonion (Figure 1A2).

Nasolabial Angle: The angle was measured between the columella, subnasale, and labiale superius (Figure 1A1).

Nasolabial Fold Angle: The angle was measured between the highest point of the nasolabial fold and the alar rim. Measurements were taken for both the right and left sides (Figure 1C1 and 1C2).

### 2.6. Statistical analysis

The Statistical Package for the Social Sciences 22 package program was used for statistical analysis, and statistically significant degrees were considered as *P* < .05. Skewness and kurtosis statistics were utilized to assess the normality of the data distribution. Values falling between +1.5 and −1.5 were considered to indicate a normal distribution. Based on these results, which confirmed that the data followed a normal distribution, a parametric test was applied to evaluate the differences between pre-and post-treatment measurements. For comparing continuous variables between two groups, the Student *t* test was used to meet the assumptions of the statistical hypotheses. Where mean, standard deviations and minimum-maximum values summarized relevant, continuous variables. Moreover, histograms were utilized for the graphical representation of categorical variables. Histograms and scatter plots with regression lines were also used to illustrate the effect of the applied formula across different observation groups.

## 3. Results

This study evaluated the effects of hyaluronic acid filler application to the temporal and zygomatic areas on the rejuvenation of the glabella and nasolabial regions in 31 women aged 35 to 55, with a mean age of 45.97 ± 7.10.

### 3.1. Glabella region

#### 3.1.1. Treatment effect

**Figure 2.**
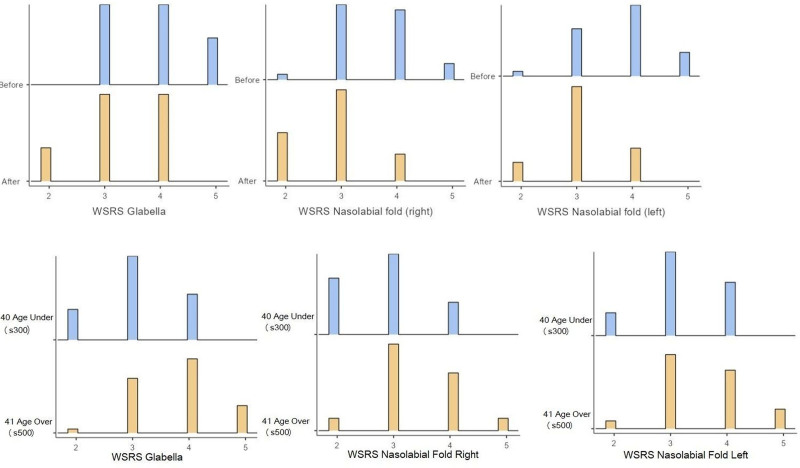
General distribution of WSRS values in the glabella and nasolabial regions and WSRS values in the glabella and nasolabial regions according to age. WSRS = Wrinkle Severity Rating Scale.

The impact of the treatment was assessed using the WSRS (Wrinkle Severity Rating Scale) parameter. A significant reduction in wrinkles in the glabella region was observed, especially 12 weeks after the application (*P* = .004) (Table 1; Fig. 2). This indicates that hyaluronic acid injections into the temporal and zygomatic regions positively affect glabella wrinkles at 4 weeks and 12 weeks after application.

**Table 1:** The distribution of measurements before treatment, at 4 weeks, and at 12 weeks post-treatment.

Parameters	Before Mean ± SD (Max–Min)	After (4 weeks) Mean ± SD (Max–Min)	After (12 weeks) Mean ± SD (Max–Min)	*P*
Nasolabial angle	92.76 ± 12.85 (64.13–114.27)	92.49 ± 11.04 (65.00–113.00)	91.49 ± 13.04 (64.00–113.00)	.423
Nasolabial fold angle (right)	20.72 ± 5.44 (11.90–36.11)	20.04 ± 3.52 (11.01–28.03)	18.04 ± 4.52 (11.31–28.93)	.039
Nasolabial fold angle (left)	20.92 ± 5.70 (11.00–32.03)	20.76 ± 4.38 (10.48–29.24)	18.76 ± 4.68 (12.37–28.24)	.108
Facial convexity	168.84 ± 5.49 (160.16–179.41)	168.12 ± 7.68 (140.95–178.03)	167.12 ± 8.69 (130.75–179.73)	.355
Total facial convexity	136.84 ± 5.67 (125.04–148.01)	136.56 ± 4.47 (124.71–146.54)	136.26 ± 5.47 (125.00–147.74)	.682
Facial concavity	166.51 ± 5.55 (156.67–176.69)	166.28 ± 5.13 (152.78–173.27)	165.68 ± 6.03 (149.78–176.27)	.572
WSRS glabella	3.84 ± 0.78 (3–5)	3.56 ± 0.66 (2–4)	3.26 ± 0.73 (2–4)	.004
WSRS nasolabial fold (right)	3.58 ± 0.72 (2–5)	3.12 ± 0.68 (2–4)	2.87 ± 0.67 (2–4)	.000
WSRS nasolabial fold (left)	3.77 ± 0.76 (2–5)	3.44 ± 0.66 (2–4)	3.10 ± 0.60 (2–4)	.000

Max = maximum, Min = minimum, *P* = significance value, SD = standard deviation, WSRS = Wrinkle Severity Rating Scale.

#### 3.1.2. Age correlation

**Figure 3.**
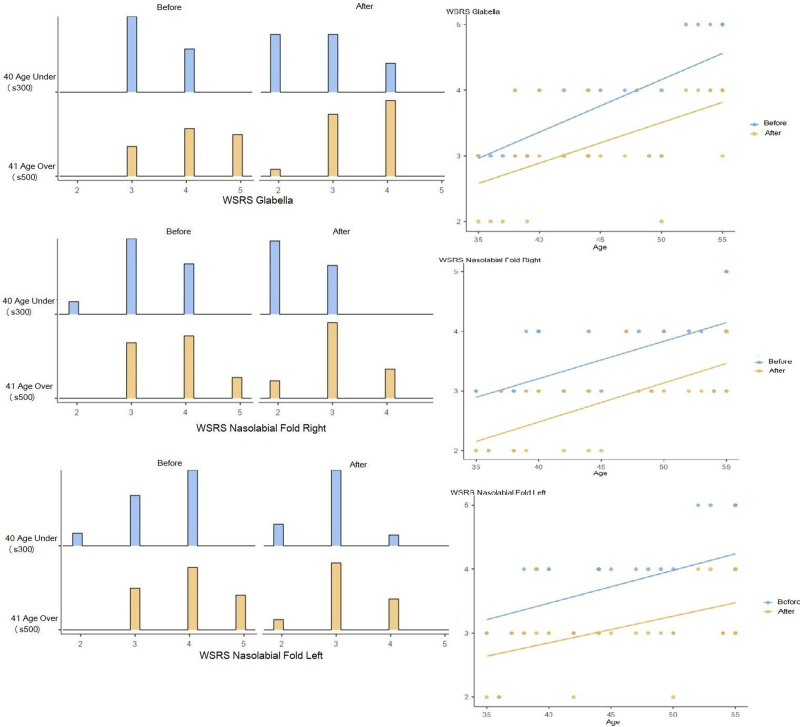
The distribution and correlation of WSRS values in the glabella and nasolabial regions according to age before and after applications. WSRS = Wrinkle Severity Rating Scale.

Analysis of the relationship between age and wrinkle improvement showed a significant increase in wrinkle improvement with advancing age (*r* = 0.601, *P* = .000). This suggests that the positive effects of hyaluronic acid on wrinkle reduction were more pronounced in older individuals (Table 2; Figs. 2 and 3).

**Table 2:** The distribution of measurements according to the density of the products used.

Parameters	S300 Mean ± SD	S500 Mean ± SD	*P*
Age	38.19 ± 2.87	49.95 ± 4.84	.000
Nasolabial angle	95.99 ± 14.61	90.15 ± 11.55	.120
Nasolabial fold angle (right)	18.55 ± 4.36	19.81 ± 5.50	.328
Nasolabial fold angle (left)	20.10 ± 4.66	19.81 ± 5.64	.831
Facial convexity	166.85 ± 6.95	168.56 ± 7.44	.376
Total facial convexity	136.53 ± 6.81	136.56 ± 4.85	.985
Facial concavity	167.32 ± 5.01	165.47 ± 6.08	.206
WSRS glabella	3.10 ± 0.70	3.78 ± 0.76	.001
WSRS nasolabial fold (right)	2.86 ± 0.73	3.41 ± 0.74	.007
WSRS nasolabial fold (left)	3.19 ± 0.68	3.56 ± 0.78	.059

*P =* significance value, SD = standard deviation, WSRS = Wrinkle Severity Rating Scale.

#### 3.1.3. S300 and S500 products

The study assessed the effects of different formulations, specifically S300 and S500. Results indicated that S300 and S500 formulations contributed to significant wrinkle reduction in the glabella area. However, S500 was associated with more pronounced results, demonstrating increased efficacy compared to S300 (Table 2; Figs. 2 and 3).

#### 3.1.4. Anatomical parameters

No significant changes were observed in anatomical parameters such as facial concavity and convexity, 4 weeks and 12 weeks after the application (Table 1).

### 3.2. Nasolabial region

#### 3.2.1. Treatment effect

The study also investigated the indirect effects of hyaluronic acid filler application on the nasolabial region. A significant reduction in WSRS scores was observed in both the right (*P* < .001) and left (*P* < .001) nasolabial folds post-treatment (Table 1; Fig. 2).

#### 3.2.2. Age correlation

Improvement in the nasolabial folds with increasing age was found to be similar to that observed in the glabella region (right side: *r* = 0.693, *P* = .000; left side: *r* = 0.496, *P* = .005) (Figs. 2 and 3).

#### 3.2.3. S300 and S500 products

The impact of S300 and S500 on the nasolabial folds was also examined. Both formulations contributed to a reduction in nasolabial fold depth, with S500 showing a more significant improvement than S300 (Table 2; Figs. 2 and 3).

#### 3.2.4. Anatomical measurements

Among the anatomical measurements of the nasolabial folds, a significant improvement was noted only in the right nasolabial fold angle (*P* = .039), indicating a more pronounced effect in the right nasolabial region (Table 1).

This study demonstrated that hyaluronic acid filler application effectively improved wrinkles in the glabella region, with increased efficacy observed in older participants and those using the S500 formulation compared to the S300. While no significant changes were observed in facial concavity and convexity, notable indirect benefits were seen in the nasolabial folds, particularly with the S500 formulation showing more pronounced effects.

We hypothesized that applying hyaluronic acid fillers to the temporal and zygomatic regions could provide a beneficial secondary effect on the glabellar and nasolabial areas. Our findings supported by statistical evidence this hypothesis.

The results in the glabellar region showed that hyaluronic acid injections into the temporal and zygomatic areas significantly reduced wrinkles, especially 12 weeks after application (*P* = .004), indicating a positive impact on glabellar wrinkles through indirect treatment. This supported our hypothesis that hyaluronic acid fillers could effectively affect the glabellar region indirectly. Additionally, the correlation between age and wrinkle improvement (*r* = 0.601, *P* = .000) suggested that hyaluronic acid treatment had more pronounced effects in older individuals, reinforcing that indirect applications could have been particularly beneficial in this group.

In the nasolabial region, the results also aligned with our hypothesis, showing a significant reduction in the depth of nasolabial folds, both on the right (*P* < .001) and left (*P* < .001) sides post-treatment. The improvement observed in nasolabial folds with increasing age (right: *r* = 0.693, *P* = .000; left: *r* = 0.496, *P* = .005) mirrored the findings in the glabellar region, indicating that the indirect effects of hyaluronic acid fillers extended beyond the glabellar region to the nasolabial folds as well.

When we compared the effects of the S300 and S500 formulations, both contributed to wrinkle reduction in both regions, but the S500 formulation demonstrated more pronounced results, showing greater efficacy than S300. This suggested that the S500 formulation might have offered more effective results in indirect treatments, supporting the strategic use of hyaluronic acid in these regions.

### 3.3. Retrospective results of physician notes

In this retrospective study, no serious complications were documented in the physician notes in individuals receiving hyaluronic acid injections in the short and long term. However, localized edema was observed in five patients immediately after the procedure; this is believed to be related to the invasive nature of the procedure, particularly related to the effects of the cannula and needle tip. In particular, localized edema resolved spontaneously in the short term.

In addition, a review of the physician notes indicated that patients’ skin has a moist and vibrant appearance after hyaluronic acid application. These positive changes were well received by patients who provided positive feedback. Our results supported the skin-enhancing effects of hyaluronic acid and suggested that the procedure could be performed safely.

## 4. Discussion

In facial aesthetics, the glabella and nasolabial regions are key areas for rejuvenation procedures. The glabella, located between the eyebrows and containing branches of the frontal nerve, is important functionally and aesthetically. Similarly, the nasolabial region, extending from the nose to the mouth corners, plays a crucial role in facial expressions. Both regions are considered anatomical danger zones, with direct rejuvenation interventions posing potential risks.^[10]^ Direct injections into the glabella can increase the risk of complications due to the sensitivity of the nerve networks and vascular structures in this area. Injections near the frontal nerve branches can lead to nerve damage, tissue necrosis, or temporary motor function loss.^[5]^ Likewise, filler applications in the nasolabial region can potentially damage deep vessels and nerves in this area. Such complications can negatively impact aesthetic results and pose significant risks.^[11]^ Given these risks, evaluating indirect methods for rejuvenation in these areas is essential. Indirect approaches aim to improve signs of aging in the glabella and nasolabial regions through interventions in adjacent areas. For instance, fillers or contouring treatments in the cheek regions can indirectly enhance the appearance of nasolabial folds and keep filler materials away from the sensitive nerve and vascular structures in the glabella.^[12]^ Literature reviews indicate that indirect rejuvenation methods can reduce the risk of complications while effectively improving aesthetic outcomes. Specifically, fillers applied to the lower and mid-face can indirectly address aging signs in the upper face and eye area.^[13–15]^ The literature not only includes studies where hyaluronic acid has been directly applied to the nasolabial and glabellar regions with reported outcomes, but also presents cases highlighting the complications observed in these areas following such procedures.^[16]^ A case reported in the literature involves nasal skin necrosis following hyaluronic acid filler injection for nasolabial fold (NLF) enhancement. Despite the injection procedure being performed carefully, complications emerged rapidly. In the 34-year-old patient, this complication triggered a vascular event, leading to the development of nasal skin necrosis.^[17]^ Similarly, a 36-year-old previously healthy female who underwent hyaluronic acid injection for cosmetic purposes into the nasolabial folds was diagnosed with arterial compromise due to vascular occlusion.^[18]^ Another study reported cases where vision loss, ptosis, ophthalmoplegia, periocular pain, nausea, skin changes in the glabellar and forehead regions, and sensory impairment in the left maxillary branch dermatome (V2) occurred following hyaluronic acid injections.^[4]^ The complications reported in the literature have prompted us to explore indirect hyaluronic acid applications improving the nasolabial fold and glabellar regions as an alternative approach to minimize the risks associated with direct treatments in these areas. Techniques such as aspiration and the use of cannulas, as suggested by Xisong et al,^[19]^ may enhance safety during direct glabella injections. However, given the associated risks, our study highlights that indirect approaches represent a safer and more effective alternative. Additionally, cadaver studies investigating intraoral injection techniques have demonstrated that this method is both safe and anatomically advantageous for treating the nasolabial area.^[20]^ Our findings support the view that such low-risk, targeted techniques may serve as an effective option, particularly in the treatment of nasolabial folds.

Our study evaluated the evaluated the effects of hyaluronic acid injections in the zygomatic and temporal regions on facial angles, using the glabella as a primary reference point to assess facial concavity and convexity. However, one month after the procedure, we did not observe statistically significant changes in facial concavity and convexity, both at 4 weeks and 12 weeks post-procedure. This might indicate that a hyaluronic acid volume greater than 4 mL might be necessary, that the use of high-density filler types such as e.p.t.q. s500 might be more appropriate, or a larger participant sample may be needed to achieve statistical significance. Additionally, as the literature indicates, combination therapies or surgical interventions may offer more effective results regarding facial angles and shape changes. Hyaluronic acid fillers can be a viable alternative for patients reluctant to undergo surgical procedures. The literature demonstrates that hyaluronic acid is an effective alternative for facial shaping and is among the minimally invasive methods. However, optimal results may require hyaluronic acid treatments to be complemented by combination therapies, high-density fillers (e.p.t.q. s500), or evaluation with larger datasets. Therefore, more comprehensive studies are needed to enhance the efficacy of hyaluronic acid applications in facial shaping and to identify the most suitable treatment options for patients.

Our findings supported the hypothesis that hyaluronic acid fillers applied to the temporal and zygomatic regions could produce beneficial secondary effects on the glabellar and nasolabial areas. In the glabellar region, hyaluronic acid injections significantly reduced wrinkles, particularly 12 weeks post-treatment (*P* = .004), highlighting the positive impact of indirect treatment. The correlation between age and wrinkle improvement (*r* = 0.601, *P* = .000) further suggested that older individuals might experience more pronounced benefits, emphasizing the value of indirect applications for this group. Similarly, in the nasolabial region, significant reductions in fold depth were observed on both sides (right: *P* < .001; left: *P* < .001), with age-related improvements mirroring those in the glabellar area. When comparing the S300 and S500 formulations, both reduced wrinkles, but the S500 demonstrated superior efficacy, indicating its potential for more effective indirect treatments. These results reinforced the strategic advantage of using hyaluronic acid fillers in these regions, offering targeted, lower-risk interventions. Although both S300 and S500 formulations effectively reduced wrinkle severity, our findings suggest that S500 was more effective, particularly in older patients. This difference may be attributed to the differences between the two formulations, with factors such as viscosity and molecular structure potentially explaining the observed variation. S500 had a higher viscosity, which can provide more support to the skin, resulting in more sustained wrinkle reduction. Additionally, the molecular structure of S500 might help achieve better integration and longer-lasting results in areas such as the nasolabial fold and glabella.

Moreover, our study found a noticeable reduction in the angles of the nasolabial fold regions one month after the procedure, though this reduction was not statistically significant. This might have been due to a small sample size or insufficient dosage. While a statistically significant reduction was observed in the right nasolabial fold angle, a reduction in the left nasolabial fold angle was noted. However, it was insignificant, suggesting potential anatomical or procedural differences between the right and left sides.

Additionally, results from the nasolabial and glabella wrinkle scales demonstrated significant improvement in the depth of the nasolabial fold and the glabella region one-month post-procedure. This improvement was particularly notable in individuals aged 41 and older who received e.p.t.q. s500 treatment. This indicates that as wrinkles increase with age, the procedure, using e.p.t.q. s300 and s500, reduced wrinkles in both age groups. These findings suggested that e.p.t.q. s500 provided a more effective improvement in the glabella and nasolabial fold regions.

The efficacy of hyaluronic acid fillers in improving the appearance of nasolabial folds and the glabella region is well-documented in the literature. Studies by Wang et al have shown that hyaluronic acid filler applications significantly reduced the depth of nasolabial folds in the short term. In this study, conducted with 75 Asian female volunteers, effects were observed over a 3-month follow-up period, and similar improvements were also observed in our study, which reinforces the effectiveness of hyaluronic acid fillers in reducing the severity of nasolabial folds.^[21]^ These findings were consistent with broader literature highlighting the volume-enhancing potential of hyaluronic acid fillers in the mid-face region.

However, it should not be overlooked that direct filler injections, despite their promised aesthetic improvements in these regions, carry significant risks. Studies, including those by Xisong et al and Kim et al, had reported serious complications, such as vascular occlusion, tissue necrosis, and even blindness, following filler injections in the glabella and nasolabial fold regions.^[19,22]^ Specifically, the glabella area houses important anatomical structures, such as the ophthalmic artery, supraorbital artery, and supratrochlear artery, and incorrect filler placement can lead to severe complications. These serious side effects have led to a decline in the popularity of hyaluronic acid injections in the glabella region. Therefore, there was limited information in the literature regarding the efficacy of hyaluronic acid fillers in the glabella. Given these risks, there is a consensus that innovative and safer methods should be preferred, particularly those offering lower risks compared to direct filler injections.

Our study focused on the effects of filler applications on the glabella and nasolabial folds. This study investigated the efficacy of hyaluronic acid fillers applied through indirect interventions using fillers in the zygomatic and temporal regions. This indirect approach allows aesthetic improvements in these regions without direct injections in high-risk areas. The benefits of direct methods are also supported by studies such as those by Xie et al In this study, the use of different filler densities resulted in significant improvements in facial wrinkles, with effects lasting 6 to 12 months.^[23]^ In our study, two different hyaluronic acid filler types, S300 and S500, were compared. However, it was observed that the S500 formulation offered longer-lasting effects. Additionally, another study has found positive results from combination therapies, such as micro-focused ultrasound and light therapy, in treating nasolabial folds, further reinforcing the efficacy of indirect methods.^[24]^

In clinical practice, the findings of this study emphasize the importance of indirect methods, considering the anatomical risks associated with direct hyaluronic acid injections. It is recommended that clinicians avoid direct injections in high-risk areas, such as the glabella and nasolabial folds, by using hyaluronic acid fillers in the zygomatic and temporal areas. Indirect methods continue to provide aesthetic improvements while minimizing the risk of complications. When selecting fillers, clinicians should consider individual patient needs, including factors such as age, skin type, and treatment goals. In our study, the S500 formulation, with its higher viscosity, was observed to provide more effective and longer-lasting results, suggesting that S500 may be a more suitable option, especially for older patients.

Another important aspect is the evaluation methods used to assess the efficacy of hyaluronic acid fillers. The literature typically relies on scale-based measurements. However, in our study, we used objective morphometric measurements to evaluate the efficacy of filler applications. Objective morphometric measurements provide more precise and accurate results, helping to prevent potential biases in the assessments. Particularly in scale-based evaluations, observational biases and subjective perceptions may have an impact. Therefore, objective measurements allowed us to assess filler efficacy more reliably and contributed to obtaining more accurate results. We emphasize the importance of using such objective measurements more widely in evaluating the clinical efficacy of filler applications.

These results suggested that age and the form and density of the filler used were critical factors in the healing process for the nasolabial folds and glabella regions, and they play important roles in achieving more effective improvement. In this study, we acknowledged the limitations of our research, including the small sample size and the retrospective design. The small sample size may limit our findings’ generalizability and reduce the analysis’s statistical power. Additionally, the study’s retrospective nature introduces potential biases, such as selection bias and reliance on historical data, which could affect the reliability of the results. To address these limitations, future research should focus on larger, prospective cohorts to provide more robust and conclusive data. Such studies would enable a deeper understanding of the effects of hyaluronic acid filler treatments on the nasolabial folds and glabella regions, considering factors such as age, filler type, and other variables.

## 5. Conclusion

This study significantly contributes to understanding the efficacy and potential of hyaluronic acid in anti-aging treatments across various age groups. The research provides valuable insights into aesthetic dermatology applications by scientifically analyzing the role of hyaluronic acid in reducing age-related wrinkles and the depth of nasolabial folds. Furthermore, a detailed investigation of the glabella and nasolabial folds allows for the development of more targeted and effective treatment strategies personalized to each patient’s specific needs. Clinically, this study highlights the potential of hyaluronic acid as a safe and effective tool for improving facial aesthetics, offering a foundation for clinicians to optimize treatment approaches, minimize risks, and enhance outcomes for individuals seeking noninvasive facial rejuvenation. As a result, it lays the groundwork for future research and clinical practices aimed at refining hyaluronic acid-based treatments to achieve more precise and lasting aesthetic results. Future research should focus on larger, prospective trials to further validate the efficacy and safety of indirect hyaluronic acid applications in facial rejuvenation.

## Acknowledgments

We would like to thank to JETEMA Co., Ltd. (https://www.jetema.com/) for all important contributions. Also, we would like to thank to Çukurova University Scientific Research Projects Unit (project number TSA-2024-16787).

## Author contributions

**Conceptualization:** Ayse Gul Kabakci, Memduha Gülhal Bozkir.

**Data curation:** Ayse Gul Kabakci, Çağlar Cengizler, Eda Esra Esen, Yaşam Türközer.

**Formal analysis:** Ayse Gul Kabakci, Çağlar Cengizler.

**Funding acquisition:** Ayse Gul Kabakci.

**Investigation:** Ayse Gul Kabakci, Eda Esra Esen, Yaşam Türközer.

**Methodology:** Ayse Gul Kabakci, Dilek Eren, Dursun Murat Bozkir.

**Project administration:** Memduha Gülhal Bozkir.

**Resources:** Ayse Gul Kabakci, Dilek Eren, Dursun Murat Bozkir.

**Software:** Ayse Gul Kabakci, Çağlar Cengizler.

**Supervision:** Ayse Gul Kabakci, Memduha Gülhal Bozkir.

**Validation:** Çağlar Cengizler.

**Writing – original draft:** Ayse Gul Kabakci.

**Writing – review & editing:** Ayse Gul Kabakci.
